# Quantum-enhanced multiparameter estimation in multiarm interferometers

**DOI:** 10.1038/srep28881

**Published:** 2016-07-06

**Authors:** Mario A. Ciampini, Nicolò Spagnolo, Chiara Vitelli, Luca Pezzè, Augusto Smerzi, Fabio Sciarrino

**Affiliations:** 1 Dipartimento di Fisica, Sapienza Università di Roma, Piazzale Aldo Moro 5, I-00185 Roma, Italy; 2 QSTAR, INO-CNR and LENS, Largo Enrico Fermi 2, I-50125 Firenze, Italy

## Abstract

Quantum metrology is the state-of-the-art measurement technology. It uses quantum resources to enhance the sensitivity of phase estimation over that achievable by classical physics. While single parameter estimation theory has been widely investigated, much less is known about the simultaneous estimation of multiple phases, which finds key applications in imaging and sensing. In this manuscript we provide conditions of useful particle (qudit) entanglement for multiphase estimation and adapt them to multiarm Mach-Zehnder interferometry. We theoretically discuss benchmark multimode Fock states containing useful qudit entanglement and overcoming the sensitivity of separable qudit states in three and four arm Mach-Zehnder-like interferometers - currently within the reach of integrated photonics technology.

Quantum metrology exploits particle entanglement in the probe state to enhance the precision of parameter estimation beyond what is reachable with classical resources (see refs 1,2 for reviews). The role of particle entanglement in the estimation of a single parameter has been clarified^3^,^4^,^5^,^6^ and investigated experimentally in Mach-Zehnder interferometers (MZIs)^7^. However, much less is known about the role of particle entanglement in the joint estimation of multiple parameters. Multiparameter estimation is relevant in many practical applications, including quantum imaging^8^, quantum process tomography^9^, as well as probing of biological samples^10^. Interestingly, the theory of multiphase estimation does not follow trivially from what is known about the single parameter case^11^,^12^. Indeed, ultimate multiphase estimation bounds are not saturable in general^13^, due to the non-commutativity of the operators generating the phase shift transformations^14^,^15^. First insights on this scenario have been recently reported^16^,^17^,^18^,^19^,^20^,^21^,^22^.

A natural platform for multiparameter quantum metrology is provided by multiport interferometry, generalizing conventional two-mode interferometry. Recent progresses in the realization of multiport devices have been achieved by exploiting integrated photonics^23^,^24^,^25^,^26^,^27^,^28^,^29^,^30^,^31^. Three- and four-port beam-splitters (tritters and quarters) have been produced with integrated optics^31^,^32^,^33^,^34^. This paves the way toward the realization of multiarm interferometers created by two tritters (quarters) in succession^35^. Quantum-enhanced single parameter estimation in integrated interferometers has been theoretically predicted^17^, while multiparameter estimation in multi-arm interferometers has been examined and compared with the sensitivity achievable by multiple single-parameter estimation^18^.

In this manuscript we provide conditions of useful particle entanglement for the simultaneous estimation of multiple phases. We study a general multimode scenario where each particle is treated as a qudit. Furthermore, we adapt the theory to the case of multiarm Mach Zehnder interferometers (MMZIs) considering an experimentally relevant framework, with multiphoton Fock states as probe and photon counting measurement. Our analysis generalizes the case of twin-Fock MZI which has attracted large experimental^7^,^36^,^37^,^38^ and theoretical^39^,^40^,^41^ interest for quantum-enhanced single phase estimation. From the analysis of the Fisher information and employing an adaptive multiphase estimation, we predict a multiparameter estimation sensitivity beyond the limit achievable with separable qudit probe states.

## Results

### Multiparameter estimation

**Figure 1:**
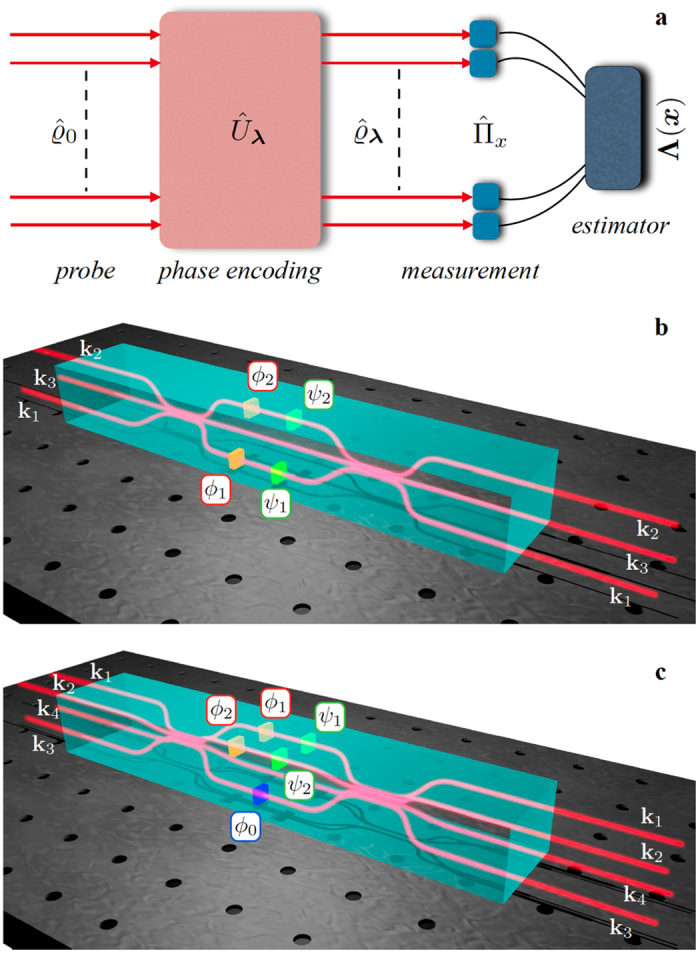
(**a**) General scheme of multiparameter estimation. (**b**) Three-mode MMZI for two-parameter phase estimation which can be obtained by two cascaded three-port beam-splitters. Phases (*ϕ*_1_, *ϕ*_2_) on modes (**k**_1_, **k**_2_) are the parameters to be estimated, while (*ψ*_1_, *ψ*_2_) are two additional controlled phase-shifts (**c**). Four-mode interferometer for two-parameter phase estimation which can be obtained by two cascaded four-port beam-splitters. Phases (*ϕ*_1_, *ϕ*_2_) on modes (**k**_1_, **k**_2_) are the parameters to be estimated, while (*ϕ*_0_, *ψ*_1_, *ψ*_2_) are assumed known and controlled. Controlled phases are introduced for adaptive estimation schemes.

We consider here the estimation of a *n*-dimensional vector parameter ***λ*** = (*λ*_1_, 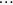, *λ*_*n*_)^11^,^12^. In our benchmark, every parameter corresponds to a phase to be estimated in a multiarm interferometer. A general approach (see Fig. 1a) consists in preparing a probe state 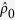, applying a ***λ***-dependent unitary transformation 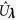 and performing independent measurements on *ν* identical copies of the output state 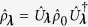. The measurement is described by a positive-operator valued measure (POVM), i.e. a set 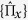 of positive operators satisfying 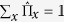, 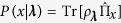 being the probability of the detection event *x*. Finally, the sequence ***x*** ≡ (*x*_1_, 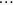, *x*_*ν*_) of *ν* measurement results is mapped into a vector parameter **Λ**(***x***) = (Λ_1_(***x***), 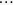, Λ_*n*_(***x***)), representing our estimate of ***λ***. A figure of merit of multiparameter estimation is the covariance matrix


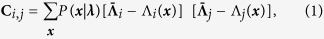


where 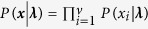 and 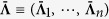 is the mean value of the estimator vector. For locally unbiased estimators (i.e. 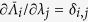) the covariance matrix is bounded, via the Cramer-Rao theorem^11^, as


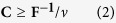


(in the sense of matrix inequality), where


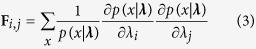


is the Fisher information matrix (FIM). Notice that Eq. (2) can be derived only when the FIM is invertible. The equality sign in Eq. (2) is saturated asymptotically in *ν* by the maximum likelihood estimator^11^. Here we quantify the phase sensitivity by the variance of each estimator, (*δλ*_*j*_)^2^ ≡ ***C***_*j*,*j*_. We have


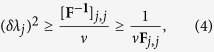


where the first inequality is due to (2) and the second follows from a Cauchy-Schwarz inequality (see Supplementary Information). Since 1/(*ν***F**_*j*,*j*_) is the Cramer-Rao bound for single parameter estimation, inequality (4) tells us that sensitivity in the estimation of *λ*_*j*_ can be optimized when fixing all the other parameters to known values. We will also consider


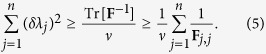


The right-hand side inequality in Eqs (4) and (5) is saturated if and only if the FIM is diagonal. Furthermore, the FIM is bounded by the quantum Fisher information matrix (QFIM): **F** ≤ **F**_**Q**_ (in the sense of matrix inequality), where


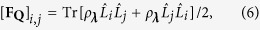


and 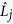 is the symmetric logarithmic derivative of *ρ*_***λ***_ with respect to parameter *λ*_*j*_, defined by 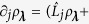

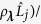
2 10. In the single parameter case, the QFIM reduces to a single scalar quantity and it is always possible to find a POVM for which *F* = *F*_*Q*_ and *δλ* = 1/*F*_*Q*_ holds^42^,^43^. In contrast, in the multiparameter case, it is generally not possible to achieve the Cramer-Rao bound^13^,^14^,^15^.

### Sensitivity bounds for qudit-separable states

In the following we consider the estimation of *n* parameters in a system made of *d* = *n* + 1 modes (e.g. the number of arms in a MMZI, see below). A single particle occupying the *n* + 1 modes is generally indicated as a qudit. The notion of qudit generalizes the concept of qubit (a two-mode particle, *n* = 1) and is relevant in multimode interferometry^2^. Here we set sensitivity bounds for multiparameter estimation when the probe state is qudit-separable. A state 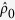 of *N* qudits is said to be qudit-separable if it can be written as 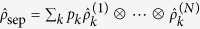, where 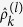 (*l* = 1, 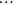, *N*) is a single qudit state, *p*_*k*_ > 0 and ∑_*k*_ *p*_*k*_ = 1. A state which is not qudit-separable is qudit-entangled. We take the generator of each phase shift, 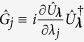 (*j* = 1, …, *n* labels the parameter), to be local in the qudit, *i.e*. it can be written as 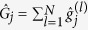 where 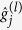 is an arbitrary operator acting on the *l*th qudit. In particular, the transformation 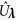 does not create entanglement among the *N* qudits. For simplicity, we will take the same operator 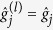 for each particle. For a generic separable probe state 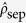, the inequality


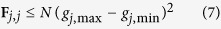


holds for all possible POVMs (see Supplementary Information), where *g*_*j*,max_ and *g*_*j*,min_ are the maximum and minimum eigenvalue of 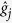, respectively. Inequality (7) gives a bound on the diagonal elements of the FIM. It corresponds, via the inequality (*δλ*_*j*_)^2^ ≥ 1/*ν***F**_*j*,*j*_, to a bound on the sensitivity reachable with qudit-separable states for the estimation of the single parameter *λ*_*j*_, when all other parameters are set to zero. Inequality (7) can be always saturated by optimal states and measurements (see Supplementary Information). For the estimation of a single parameter, the violation of Eq. (7) is a necessary and sufficient condition of useful qudit entanglement^2^,^4^: only those qudit-entangled states that violate Eq. (7) allow to estimate the parameter *λ*_*j*_ with a sensitivity overcoming the one reachable with any qudit-separable state. Regarding the simultaneous estimation of multiple parameters, we can use Eq. (7) and the chain of inequalities (4) to obtain


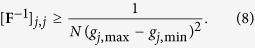


Inequality (8) is a bound of sensitivity in the estimation of the single parameter *λ*_*j*_ with qudit-separable states, when all the parameters are unknown. Summing Eq. (8) over all parameters, we obtain


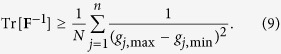


According to Eqs (8) and (9), for qudit-separable states such that the FIM is invertible, we recover – at best – the shot noise scaling of phase sensitivity, *δλ*_*j*_ ∝ *N*^−1/2^, which also characterizes single parameter estimation^3^,^4^. Notice that the quantity (*g*_*j*,max_ − *g*_*j*,min_)^2^ is equal to one for any qubit transformation and might be larger than one for general qudit transformations. We finally recall that the phase estimation scenario we are considering – as well as the notion of useful qudit-entangement – refers to interferometric scheme involving liner qudit transformations and multiple independent measurements done with identical copies of the same probe. Inequalities (8) and (9) have no concern with the qudit-entanglement of the initial probe state for (nonlinear) parameter dependent processes that entangle/disentangle the probe or non-independent multiple measurements.

### Multimode Mach-Zehnder interferometry

In the following we discuss the estimation of a phase vector ***ϕ*** = (*ϕ*_1_, …, *ϕ*_*n*_) in a MMZI (see Fig. 1b,c). The MMZI can be obtained by cascading a *d*-mode balanced beam-splitter 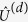, a phase shift transformation 
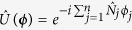
, being 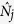 the photon-number operator for the *i*th mode, and a second multiport beam-splitter 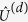. The *d*-mode beam-splitter 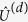 is the natural extension of the standard 50-50 beam-splitter to more than two optical input-output modes^41^. Hence, the MMZI can be adopted as a benchmark to investigate simultaneous estimation of *n* = *d* − 1 optical phases. Indeed, it allows for a direct comparison between classical and quantum probe states and represents a flexible platform for the analysis of multiparameter scenario by changing the unitary transformation of the input and output multiport beam-splitters.

In order to adapt the discussion of the previous section, we consider *N* particles as input of the MMZI and identify a single particle in the *d* arms of the interferometer as a qudit, whose Hilbert space has thus dimension *d*. The generator of phase shift in the *j*th mode is 
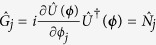
. One can thus write 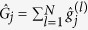 where 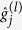 as the operator projecting the *l*th qudit on the *j*th mode. Finally, *g*_*j*,max_ − *g*_*j*,min_ = 1 and the inequalities (8) and (9) read


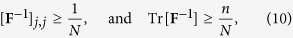


respectively. The violation of one of these inequalities in the MMZI is a signature of useful qudit-entanglement in the probe state.

The recent experimental implementation of symmetric multiport beam-splitting^31^,^32^,^33^,^34^, by adopting integrated platforms, paves the way toward the future realization of optical MMZIs. For *d* = 3 modes, the tritter matrix 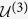, corresponding to its unitary transformation 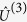, has diagonal elements 
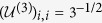
 and off-diagonal elements 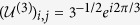 with *i* ≠ *j*. For *d* = 4 modes, the quarter matrix 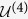 is 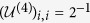 and 
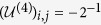
 for *i* ≠ *j*. The overall matrix for the MMZI is then obtained as 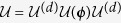. The phase vector is estimated from the measurement of the number of particles in each mode. As probe, we focus on multimode Fock states with a single photon in each input mode of the interferometer^18^, |1, 1, 1〉 and |1, 1, 1, 1〉 for the three- and four-mode MZI, respectively. Here, |1, 1, 1〉 ≡ |1〉_1_ ⊗ |1〉_2_ ⊗ |1〉_3_ (and analogous definition for |1, 1, 1, 1〉), where |1〉_*j*_ is a Fock state identifying a single particle in the *j*th mode.

**Figure 2:**
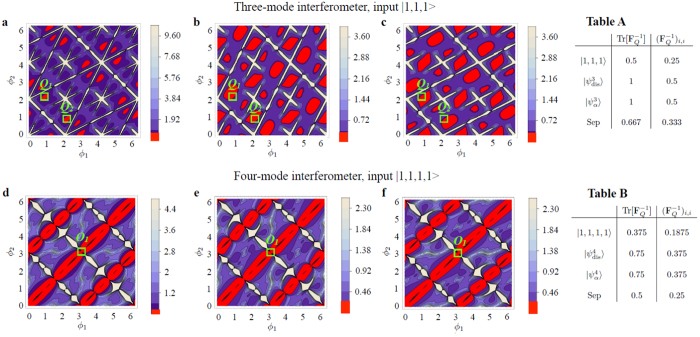
(**a**–**c**) Optimal phase sensitivity of the three-mode balanced MZI with |1, 1, 1〉 probe state and photon-number measurement. Contour plots of (**a**) Tr[**F**^−1^], (**b**) (**F**^−1^)_1,1_, (**c**) (**F**^−1^)_2,2_, as a function of *ϕ*_1_ and *ϕ*_2_. Tr[**F**^−1^] is minimized at the working points *Q*_1_ and *Q*_2_ (see main text). (**d–f**) Optimal phase sensitivity of the four-mode balanced MZI with |1, 1, 1, 1〉 probe state and photon-number measurement. Contour plots of (**d**) Tr[***F***^−1^], (**e**) (**F**^−1^)_1,1_, (**f**) (**F**^−1^)_2,2_, as a function of *ϕ*_1_ and *ϕ*_2_. These are shown for *ϕ*_0_ = 0.01 to avoid undetermined points in the plot. The QCRB is achieved, for instance, at the point *O*_1_ = [*π*, *π*]. Red areas indicate the violation of the separable bound defined in Eq. (10). Tables **A** and **B** report 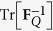 and 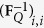 for different input states and their comparison with the separable bound (Sep).

For the three-mode MZI, the results of the calculation for **F**^−1^ are shown in Fig. 2a–c. Analytic expression of the FIM is reported in the Supplementary Information. We observe that Tr[**F**^−**1**^] and the diagonal elements [**F**^−**1**^]_1,1_ and [**F**^−**1**^]_2,2_ depend on the phases *ϕ*_1_ and *ϕ*_2_. Notably, the inequalities (10) are violated at certain optimal values of the parameters, signaling that the Fock state |1, 1, 1〉 contains useful qudit entanglement: we find 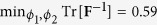 (see Fig. 2a) and 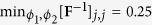 (see Fig. 2b,c), which are smaller than the bound for qudit-separable states Tr[**F**^−1^] = 0.667 and [**F**^−1^]_*j*,*j*_ = 0.33 (here *N* = 3 and *n* = 2), respectively. Additionally, we observe characteristic features. (i) ***F*** ≠ ***F***_*Q*_, in particular, the minimum value of Tr[**F**^−1^] is greater than the corresponding minimum value of the QFIM: 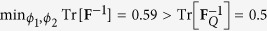 (see Fig. 2a). (ii) The FIM is not always invertible: at the phase values for which det **F** = 0 the bound (2) is not defined. Around these points (white regions in Fig. 2a–c) [**F**^−**1**^]_1,1_ and/or [**F**^−**1**^]_2,2_ diverge. (iii) The working points to obtain the minimum of the multiparameter bound do not lead to symmetric errors on the single parameters *ϕ*_1_ and *ϕ*_2_. More specifically, when Tr[**F**^−1^] = 0.59, the bounds for the error on the single parameters are different: 
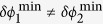
. This is obtained for instance for working point *Q*_1_ = (*ϕ*_1_,*ϕ*_2_) = (0.892, 2.190), leading to 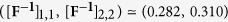 and for working point *Q*_2_ = (*ϕ*_1_,*ϕ*_2_) = (2.190, 0.892), leading to 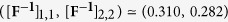, see Fig. 2a. In summary, with this choice of probe state and measurement it is not possible to saturate the quantum Cramer-Rao inequality simultaneously for the two parameters. Furthermore, according to point (iii) an adaptive estimation strategy (which we discuss below) is necessary to obtain the minimum sensitivity on both parameters with symmetric errors, and thus saturate the multiparameter Cramer-Rao bound.

We have repeated the above analysis for the four-mode interferometer (*d* = 4) with two unknown phases, *ϕ*_1_ and *ϕ*_2_, and a known control phase *ϕ*_0_ (see Fig. 1c). This configuration allows a comparison between three- and four-arm interferometers for the two parameter estimation. In the latter case the control phase *ϕ*_0_ gives us an additional degree of freedom. We choose as input the Fock State |1, 1, 1, 1〉. In Fig. 2d–f the results of our calculations are reported for a fixed value of *ϕ*_0_, as well as the numerical analysis of det **F**. We observe that as in the previous cases the FIM depends on the value of the parameter to be estimated. Furthermore, also in the four-mode the achievable sensitivity falls below the bound (10) for separable states: we have 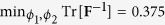, 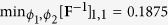 and 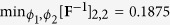 which are below the bounds 0.5 and 0.25 given by Eq. (10) (*N* = 4 and *n* = 2, here), respectively. The most notable difference with respect to the previous case is that the QCRB is achieved, for instance in working point *O*_1_ = [*π*, *π*]. In addition, both diagonal terms are equivalent and only a two step adaptive protocol is needed to reach the QCRB for any arbitrary phase vector (see discussion below).

We have also compared the obtained results with the one achievable with other probe states. For instance, we consider a set of distinguishable particles 
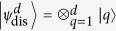
 (where |*q*〉 stands for a single photon on mode **k**_*q*_), or an input coherent state 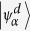 on input mode **k**_1_ with 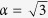 for *d* = 3 (*α* = 2 for *d* = 4) and no phase reference. We obtain 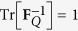 for both 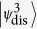 and 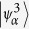, within the bound Tr[**F**^−1^] ≥ 0.667 given by Eq. (10) for separable inputs. Similarly, 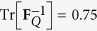 for both 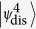 and 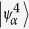, within the bound Tr[**F**^−1^] ≥ 0.5. Results are summarized in Tables A and B.

### Adaptive phase estimation

In this section we present the adaptive estimation protocols required to maximize the precision on the simultaneous estimation of two arbitrary phases in a three- and four- mode MZI. The resources (the number of independent measurements *ν*) are split between multiple steps. A first step is needed to obtain a rough estimate of the unknown phases and requires a small subset of the resources which becomes negligible when the number of repetitions *ν* of the experiment is large enough. The subsequent steps exploit the available information to optimize the estimation procedure.

**Figure 3:**
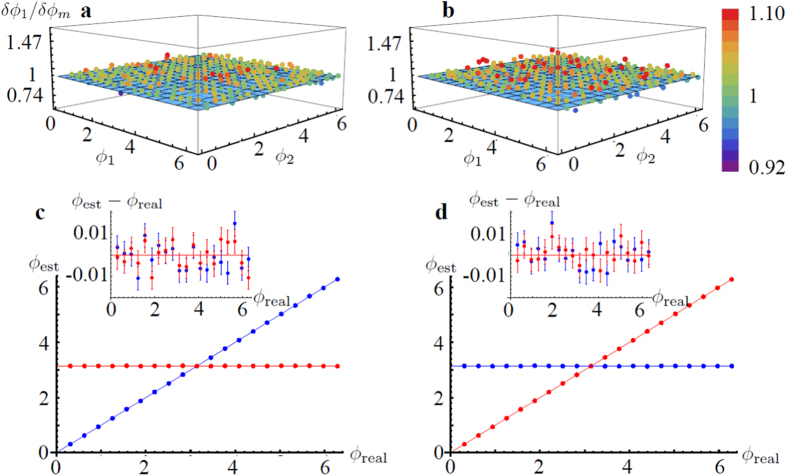
Numerical simulation of adaptive estimation of two phases, *ϕ*_1_ and *ϕ*_2_ with the three-mode interferometer injected by a |1, 1, 1〉. The adaptive protocol (see text) aims at reaching a phase uncertainty *δϕ*_1_ ≈ *δϕ*_2_ after *ν* = 10000 independent measurements. (**a**,**b**) Uncertainties *δϕ*_1_/*δϕ*_*m*_ and *δϕ*_2_/*δϕ*_*m*_ obtained for different values of *ϕ*_1_ and *ϕ*_2_ (points) and normalized with respect to the expected value *δϕ*_*m*_ = 0.543/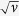 (see text). As an example, we report the results obtained for the specific cases *ϕ*_1_ = *π* (**c**) and *ϕ*_2_ = *π* (**d**). In these panels the blue line is the estimated value of *ϕ*_1_, the red line is the estimated *ϕ*_2_. The inset shows the difference between the estimated value and the actual value of the phases, error bars are obtained by repeating 1000 times the numerical simulation of the protocol.

Regarding the three-mode interferometer, the above analysis has identified working points (*Q*_1_ and *Q*_2_) where the minimum uncertainty for the estimation of the two phases *ϕ*_1_ and *ϕ*_2_ does not give the same error on the two individual parameters. To overcome this limitation – and obtain approximatively a symmetric error in the joint estimation of the two phases – we exploited a three-step adaptive algorithm. The protocol requires *ν* independent measurements and the adoption of controlled phase shifts *ψ*_*i*_ on modes **k**_*i*_, with *i* = 1, 2, which have to be tuned during the protocol to perform the estimation at different working points (see in Fig. 1b). In a first step, we set *ψ*_1,2_ = 0 and obtain a rough estimate of the phases *ϕ*_*i*_ after a number of measurements much smaller than *ν*. Then, in step 2 the tunable phases *ψ*_*i*_ are adjusted so that *ϕ*_*i*_ + *ψ*_*i*_ on arms 1 and 2 are set to be close to the working point *Q*_1_. In this step essentially half of the remaining resources are spent so as to obtain 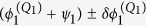 and 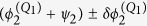 with an adequate estimator. Here 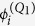, 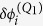 represent respectively the estimation and the uncertainty of *ϕ*_*i*_ around working point *Q*_1_. In step 3 the same procedure is repeated for working point *Q*_2_. Finally the tunable phases *ψ*_1.2_ are subtracted so to recover *ϕ*_1,2_ ± *δϕ*_1,2_. The results of the adaptive algorithm are shown in Fig. 3a–d. Half of the measurements (*ν*_1_ = *ν*/2) are performed at point *Q*_1_, where 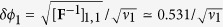 and 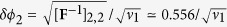, while the other half (*ν*_2_ = *ν*/2) are performed at point *Q*_2_, where 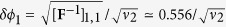 and 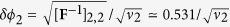. The expected error on a single phase *δϕ*_*i*_ after the two steps is then obtained as an appropriate combination of the values on the points *Q*_*i*_. More specifically, as the Fisher information is additive, the overall FIM reads **F** = *ν*_1_**F**_1_ + *ν*_2_**F**_2_, where **F**_*i*_ is the FIM in working points *Q*_*i*_. We observe that the protocol permits to achieve the bound of the working point, which for *ν*_1_ = *ν*_2_ is 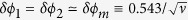. Note that the bound is lower than the bound (10) for separable states 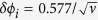.

**Figure 4:**
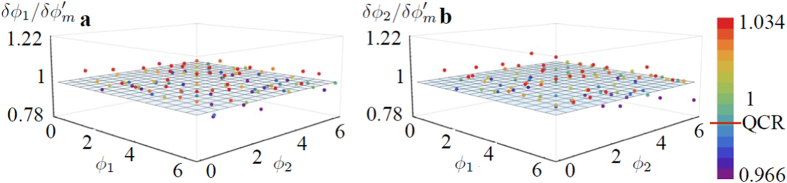
Numerical simulation of adaptive estimation of two phases, *ϕ*_1_ and *ϕ*_2_ with the four-mode interferometer injected by a |1, 1, 1, 1〉, for *ϕ*_0_ = 0.01 and *ν* = 10000 independent measurements. (**a**,**b**) Uncertainties 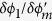 and 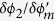 obtained for different values of *ϕ*_1_ and *ϕ*_2_ (points) and normalized with respect to the achievable bound 
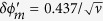
. The horizontal red line in the legend corresponds to the quantum Cramer-Rao bound for the single-parameter.

The adaptive scheme for the four-mode interferometer is slightly different: in this case there are optimal working points, as the point *O*_1_, see Fig. 2, where QCRB is achieved for both phases. To reach the QCRB for arbitrary phases, we thus apply a two-step adaptive protocol. In the first step, we obtain a rough estimate of the parameters with an initial error *δ*. Then, in the second step we apply two supplementary phases *ψ*_1_ and *ψ*_2_ to translate the working point of the protocol to the neighbourhood of *O*_1_. It should be noticed that a convergent estimation protocol in the second step requires to set *ϕ*_0_ such that the quantity Tr[**F**^−1^] has no singularities. Note that the more *ϕ*_0_ deviates from *ϕ*_0_ = 0, the larger is the regular region around *O*_1_ (see Supplementary Information). The price to pay is a slightly increasing the error in the estimation process. The value of *ϕ*_0_ has to be chosen in order to move the singularity away from a neighbourhood of *O*_1_ larger than the inital error *δ* of the first step. The results of the protocol for the four-mode case with *ϕ*_0_ = 0.01 are then shown in Fig. 4a,b. Similarly to the three-mode case, we observe that the protocol permits to achieve the bound of the working point, which is 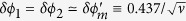 for *ϕ*_0_ = 0.01 (plane in Fig. 4), while the quantum Cramer-Rao bound reads 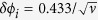. This shows that achieving a convergent numerical protocol leads to a slight decrease in phase sensitivity due to singular points in the neighborhood of the working regions. Also in this case, the adaptive protocol allows to reach a sensitivity overcoming the bound of separable state for any vector parameter.

## Conclusions

In this manuscript we have developed the general theory of quantum-enhanced multiphase estimation. In particular, we provide conditions of useful qudit-entanglement for the simultaneous estimation of multiple phases below the ultimate sensitivity limit achievable with qudit-separable states. We have focused on interferometers involving linear qudit transformations and multiple independent measurements. In a realistic experimental scenario, using multi-mode Mach-Zehnder interferometers and photo-counting measurements, Fock state probes can be exploited for multiphase estimation with quantum-enhancement phase sensitivity. With respect to the estimation of a single phase, where Fock states are known to be a useful resource, our analysis evidences a rich scenario: most notably, the phase sensitivity strongly depends on the phase value (the Cramer-Rao bound being not always definite) and on the interferometer configurations such as the three- and four-mode interferometers. Finally, we discuss and numerically simulate an adaptive estimation protocol which permits to achieve the expected bounds for any vector parameter. The adaptive strategy becomes crucial in multiparameter estimation since the simultaneous saturation of the ultimate limits for all parameters is in general not guaranteed.

During the completion of this manuscript, a first implementation of a tritter-based interferometer for single-phase estimation has been reported^45^.

## Additional Information

**How to cite this article**: Ciampini, M. A. *et al*. Quantum-enhanced multiparameter estimation in multiarm interferometers. *Sci. Rep*. **6**, 28881; doi: 10.1038/srep28881 (2016).

## Supplementary Material

Supplementary Information
