# Supplementary material for: Quantum-enhanced multiparameter estimation in multiarm interferometers
Source: Sci Rep. 2016 Jul 6;6:28881. doi: 10.1038/srep28881 (PMC4933875; doi:10.1038/srep28881)
Supplement: Supplementary Information [file srep28881-s1.pdf]

Supplementary Information: Quantum-enhanced multiparameter estimation in multiarm interferometers

Mario A. Ciampini,[1] Nicolò Spagnolo,[1,*] Chiara Vitelli,[1] Luca Pezzè,[2] Augusto Smerzi,[2] and Fabio Sciarrino[1,†]

[1] *Dipartimento di Fisica, Sapienza Università di Roma, Piazzale Aldo Moro 5, I-00185 Roma, Italy*

[2] *QSTAR, INO-CNR and LENS, Largo Enrico Fermi 2, 50125 Firenze, Italy*

SUPPLEMENTARY NOTE 1: BOUNDS ON THE DIAGONAL ELEMENTS OF THE FISHER INFORMATION MATRIX

Here we detail the demonstration of the inequality [S1]

$$[\mathbf{F}^{-1}]_{i,i} \geq \frac{1}{\mathbf{F}_{i,i}}. \tag{S1}$$

To prove Eq. (S1) we recall that the square root of the positive definite Fisher matrix is given by the matrix with the same (orthonormal) eigenvectors as $\mathbf{F}$ and the square root of its eigenvalues. We indicate as $f_i > 0$ and $\boldsymbol{v}_i$ (with $\boldsymbol{v}_i^\top \boldsymbol{v}_j = \delta_{i,j}$) the eigenvalues and eigenvectors of $\mathbf{F}$, respectively ($\mathbf{F}\boldsymbol{v}_i = f_i \boldsymbol{v}_i$). Notice that $\mathbf{F}$ is real and symmetric and thus diagonalize. In addition, $\mathbf{F}$ is positive semidefinite (therefore $f_i \geq 0$) and assuming that $\mathbf{F}$ is invertible we have $f_i \neq 0$. We write

$$\mathbf{F} = \sum_i f_i \, \boldsymbol{v}_i \boldsymbol{v}_i^\top,$$

$$\sqrt{\mathbf{F}} = \sum_i \sqrt{f_i} \, \boldsymbol{v}_i \boldsymbol{v}_i^\top,$$

$$(\sqrt{\mathbf{F}})^{-1} = \sqrt{\mathbf{F}^{-1}} = \sum_i \frac{1}{\sqrt{f_i}} \, \boldsymbol{v}_i \boldsymbol{v}_i^\top,$$

and therefore

$$\sqrt{\mathbf{F}}(\sqrt{\mathbf{F}})^{-1} = \sqrt{\mathbf{F}}\sqrt{\mathbf{F}^{-1}} = \mathbb{1},$$

with $\mathbb{1}$ the identity matrix. Using the Cauchy-Schwarz inequality

$$\begin{aligned} 1 &= \Big(\sum_k [\sqrt{\mathbf{F}}]_{i,k} [\sqrt{\mathbf{F}^{-1}}]_{k,i} \Big)^2 \\ &\leq \Big(\sum_k [\sqrt{\mathbf{F}}]_{i,k} [\sqrt{\mathbf{F}}]_{k,i} \Big) \Big(\sum_k [\sqrt{\mathbf{F}^{-1}}]_{i,k} [\sqrt{\mathbf{F}^{-1}}]_{k,i} \Big), \end{aligned}$$

we obtain

$$1 \leq \mathbf{F}_{i,i} [\mathbf{F}^{-1}]_{i,i} \qquad \forall i,$$

and recover Eq. (S1). The equality sign is saturated if and only if $\mathbf{F} = c\mathbb{1}$, where c is a positive real number.

SUPPLEMENTARY NOTE 2: UPPER BOUND TO $F_{i,i}$

The following inequality holds

$$F_{i,i} \leq F_Q^{(i)} \big[\hat{\rho}(\boldsymbol{\lambda}) \big], \tag{S2}$$

where $F_Q^{(i)} \big[\hat{\rho}(\boldsymbol{\theta}) \big] = [F_Q]_{i,i}$ is the diagonal element of the quantum Fisher information matrix,

$$F_Q^{(i)} \big[\hat{\rho}(\boldsymbol{\theta}) \big] = \mathrm{Tr}[\hat{\rho}(\boldsymbol{\lambda}) \hat{L}_i^2] \tag{S3}$$

---

* nicolo.spagnolo@uniroma1.it

† fabio.sciarrino@uniroma1.it

and $\hat{L}_i$ is the symmetric logarithmic derivative, satisfying

$$\frac{\partial \hat{\rho}(\boldsymbol{\lambda})}{\partial \lambda_i} = \frac{\hat{L}_i \hat{\rho}(\boldsymbol{\lambda}) + \hat{\rho}(\boldsymbol{\lambda}) \hat{L}_i}{2}. \tag{S4}$$

The demonstration of the inequality (S2) follows from Ref. [S2] (see also the review [S3]). Using Eqs. (7) and (S4), we have

$$\mathbf{F}_{i,i} = \sum_x \frac{1}{p(x|\boldsymbol{\lambda})} \bigg(\frac{\partial p(x|\boldsymbol{\lambda})}{\partial \lambda_i} \bigg)^2 = \sum_x \frac{\Re(\mathrm{Tr}[\hat{\rho}(\boldsymbol{\lambda}) \hat{L}_i \hat{\Pi}_x])^2}{\mathrm{Tr}[\hat{\rho}(\boldsymbol{\lambda}) \hat{\Pi}_x)]},$$

where $\Re(y)$ is the real part of y. We then use the following chain of inequalities valid for all values of x:

$$\begin{aligned} \Re\big(\mathrm{Tr}[\hat{\rho}(\boldsymbol{\lambda}) \hat{L}_i \hat{\Pi}_x]\big)^2 &\leq \big|\mathrm{Tr}[\hat{\rho}(\boldsymbol{\lambda}) \hat{L}_i \hat{\Pi}_x]\big|^2 \\ &\leq \mathrm{Tr}\big[\hat{\rho}(\boldsymbol{\lambda}) \hat{\Pi}_x\big] \mathrm{Tr}\big[\hat{\Pi}_x \hat{L}_i \hat{\rho}(\boldsymbol{\lambda}) \hat{L}_i\big], \end{aligned}$$

the first inequality due to $\Re(y)^2 \leq |y|^2$ and the second is due to Caushy-Schwarz. We thus obtain (for all values of x)

$$\frac{\Re(\mathrm{Tr}[\hat{\rho}(\boldsymbol{\lambda}) \hat{L}_i \hat{\Pi}_x])^2}{\mathrm{Tr}[\hat{\rho}(\boldsymbol{\lambda}) \hat{\Pi}_x)]} \leq \mathrm{Tr}\big[\hat{\Pi}_x \hat{L}_i \hat{\rho}(\boldsymbol{\lambda}) \hat{L}_i\big]. \tag{S5}$$

Summing over x, we thus recover Eq. (S2) with $F_Q^{(i)}\big[\hat{\rho}(\boldsymbol{\lambda})\big]$ given in Eq. (S3). The equality sign can be saturated by taking a projective measurement $\hat{\Pi}_x$ on the eigenstates of $\hat{L}_i$.

To find an explicit expression for $F_Q^{(i)}\big[\hat{\rho}(\boldsymbol{\lambda})\big]$, let us write $\hat{\rho}(\boldsymbol{\lambda})$ in diagonal form $\hat{\rho}(\boldsymbol{\lambda}) = \sum_k p_k |k\rangle\langle k|$, with $p_k \geq 0$, $\{|k\rangle\}$ is a complete basis ($\sum_k |k\rangle\langle k| = \mathbb{1}$) and $\sum_k p_k = 1$. We have

$$F_Q^{(i)}\big[\hat{\rho}(\boldsymbol{\lambda})\big] = 2 \sum_{\substack{k,k' \\ p_k + p_{k'} \neq 0}} \frac{(p_k - p_{k'})^2}{p_k + p_{k'}} \big|\langle k|\hat{H}_i|k'\rangle\big|^2,$$

where

$$\hat{H}_i \equiv i \bigg(\frac{\partial \hat{U}(\boldsymbol{\lambda})}{\partial \lambda_i} \bigg) \hat{U}^{-1}(\boldsymbol{\lambda}). \tag{S6}$$

In particular,

$$F_Q^{(i)}\big[\hat{\rho}(\boldsymbol{\lambda})\big] \leq 4 \big(\Delta \hat{G}_i\big)^2. \tag{S7}$$

The quantity $[F_Q]_{i,i}$ has the physical meaning of a single-parameter quantum Fisher information with respect to transformations $\hat{U}(\boldsymbol{\lambda})$ where all the parameters are fixed except λ_i (in other words $\frac{\partial \hat{U}(\boldsymbol{\lambda})}{\partial \lambda_j} = 0$ for $i \neq j$).

SUPPLEMENTARY NOTE 3: RELATION TO QUDIT ENTANGLEMENT

The relation between $[F_Q]_{i,i}$ and qudit entanglement is a generalisation of the criteria of useful entanglement discussed in Ref. [S4] (see also [S5] and the review [S3]). We recall that a state ϱ_0 of N qudits is said to be qudit-separable if can be written as

$$\hat{\varrho}_{\mathrm{sep}} = \sum_k p_k \, |\psi_{k,1}\rangle\langle\psi_{k,1}| \otimes |\psi_{k,2}\rangle\langle\psi_{k,2}| \otimes ... |\psi_{k,N}\rangle\langle\psi_{k,N}|,$$

where $|\psi_{k,n}\rangle$ $(n = 1, \cdots, N)$ is a single qudit state. We assume here that the Hamiltonian $\hat{G}_i$ is local in the qudits (i.e. it can be written as $\hat{G}_i = \sum_{n=1}^N \hat{g}_i^{(n)}$, where $\hat{g}_i^{(n)}$ acts on the nth qudit), and, for simplicity, we take $\hat{g}_i^{(n)} = \hat{g}_i$ for all $n = 1, \cdots, N$. Under these conditions we find

$$F_Q^{(i)} \leq N (g_{i,\max} - g_{i,\min})^2, \tag{S8}$$

where $g_{i,\max}$ and $g_{i,\min}$ are the maximum and minimum eigenvalues of $\hat{g}_i$. Taking into account Eq. (S2), we obtain that the inequality

$$F_{i,i} \leq N (g_{i,\max} - g_{i,\min})^2 \tag{S9}$$

holds for all separable qudit states and all possible POVMs. A violation of this inequality signals qudit entanglement.

The demonstration of Eq. (S8) uses general properties of the single-parameter quantum Fisher information [S3]):

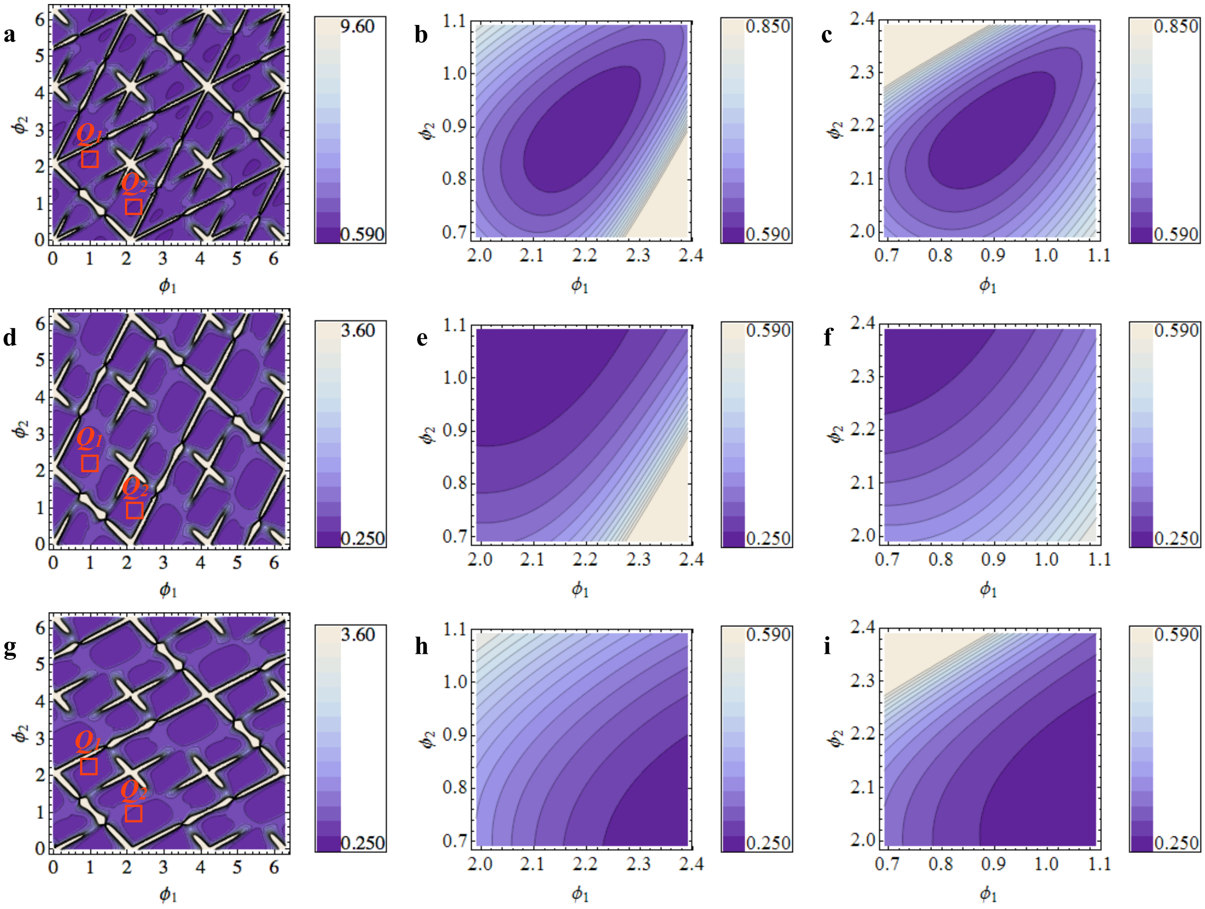

SUPPLEMENTARY FIG. S1. Phase sensitivity of the three-mode balanced MZI with $|1,1,1\rangle$ probe state and photon-number measurement. **a**, Contour plot of $\mathrm{Tr}[\mathbf{F}^{-1}]$ as a function of ϕ_1 and ϕ_2. **b-c**, Regions of $\mathrm{Tr}[\mathbf{F}^{-1}]$ around the values of the phases which minimize $\mathrm{Tr}[\mathbf{F}^{-1}]$. **d**, working point Q_1 and **c**, working point Q_2. **d**, Contour plot of $(\mathbf{F}^{-1})_{1,1}$ as a function of ϕ_1 and ϕ_2. **e-f**, Regions of $(\mathbf{F}^{-1})_{1,1}$ around the values of the phases which minimize $\mathrm{Tr}[\mathbf{F}^{-1}]$. **e**, working point Q_1 and **f**, working point Q_2. **g**, Contour plot of $(\mathbf{F}^{-1})_{2,2}$ as a function of ϕ_1 and ϕ_2. **h-i**, Regions of $(\mathbf{F}^{-1})_{2,2}$ around the values of the phases which minimize $\mathrm{Tr}[\mathbf{F}^{-1}]$. **h**, working point Q_1 and **i**, working point Q_2.

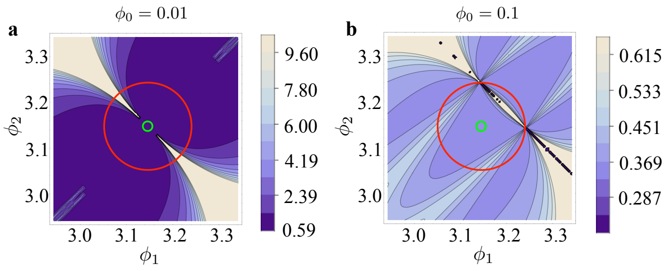

SUPPLEMENTARY FIG. S2. Analysis of stability for the four-mode interferometer with $|1,1,1,1\rangle$ probe state and photon-number measurement. To perform a two-step adaptive protocol for the estimation of two unknown phases with the highest precision, we need that in a neighbourhood of radius δ centered in the working point O_1, the quantity $\mathrm{Tr}[\mathbf{F}^{-1}]$ has no singularities. **a-b**, Contour plot of $\mathrm{Tr}[\mathbf{F}^{-1}]$ around the working point $O_1 = [\pi, \pi]$ for **a** $\phi_0 = 0.01$ and **b** $\phi_0 = 0.1$. Green circles: regions with $\delta = 0.01$ around O_1. Red circles: regions with $\delta = 0.1$ around O_1. We note that increasing ϕ_0 the singularities move away from the neighbourhood, increasing the stability of the estimation protocol.

- the convexity of $F_Q^{(i)}$:

$$F_Q^{(i)}\left[\hat{\rho}_{\mathrm{sep}}\right] \leq \sum_k p_k F_Q^{(i)}\left[|\psi_{1,k}\rangle \otimes \ldots \otimes |\psi_{N,k}\rangle\right];$$

- the additivity of $F_Q^{(i)}$:

$$F_Q^{(i)}\left[|\psi_{1,k}\rangle \otimes ... \otimes |\psi_{N,k}\rangle\right] = \sum_{n=1}^{N} F_Q^{(i)}\left[|\psi_{n,k}\rangle\right];$$

- the bound

$$F_Q^{(i)}\left[|\psi_{n,k}\rangle\right] \leq 4\big(\Delta\hat{g}_i\big)^2_{|\psi_{n,k}\rangle};$$

- the inequality

$$4\big(\Delta\hat{g}_i\big)^2_{|\psi_{n,k}\rangle} \leq \big(g_{i,\max} - g_{i,\min}\big)^2,$$

  which holds for every n, i.e. for every single-qudit state.

Putting all these results together, we arrive at Eq. (S8).

SUPPLEMENTARY NOTE 4: MODE AND PARTICLE ENTANGLEMENT

Here we discuss the role of entanglement in the section "Multimode Mach-Zehnder interferometry" of the main text, focusing on the three-mode MZI interferometer. The generalization to the four mode MZI is a trivial extension.

We first observe that the Fock state $|1,1,1\rangle = a_a^\dagger a_b^\dagger a_c^\dagger |0\rangle$ does not contain any mode entanglement, as it can be written as $|1\rangle_a \otimes |1\rangle_b \otimes |1\rangle_c$. Here $a_k^\dagger$ represent the creation operator of one particle over mode k (a, b, c represent the three spatial modes), and the notation $|1,1,1\rangle$ indicates the injection of three identical particles, one for each of the three arms of the interferometers. The particles are labeled by using the path in which they are, so that we call path notation this specific representation. Such notation is indeed very convenient as it is possible to define the tritter, quarter and phase shifter matrixes in a natural way by considering the transformation of the creation operators before and after the optical element.

Now let us define a different representation, which we call particle notation, formally equivalent to the previous one. We define now the qubit as the path taken by a particle in the interferometer, so that if the particle 1 is in mode a, we identify it as $|a\rangle_1$.

As an example, let us consider a two-particle Fock state over modes a and b. This state can be written in particle notation as:

$$|1\rangle_a|1\rangle_b \rightarrow \frac{1}{\sqrt{2}}(|a\rangle_1|b\rangle_2 + |b\rangle_1|a\rangle_2). \tag{S10}$$

Note that this two-particle wave-function is symmetric with respect to particle exchange, since we are dealing with indistinguishable photons. Eq. (S10) is clearly particle entangled in this notaton.

In the following we show the difference between particle and path representation, (see also Refs. [S6, S7]), starting from the simple case of the beam splitter trasformation, and extending it to the 3-mode case.

Beam splitter is local in particle and non local in path. The beam splitter transformation in path notation is naturally defined in terms of creation operators in the second quantization scheme:

$$b_a^\dagger \rightarrow \frac{1}{\sqrt{2}}(a_a^\dagger + a_b^\dagger), \quad b_b^\dagger \rightarrow \frac{1}{\sqrt{2}}(a_a^\dagger - a_b^\dagger). \tag{S11}$$

The Fock state can be written as $|1,1\rangle = a_a^\dagger a_b^\dagger |0,0\rangle$. Applying Eq. (S11) to the input state generates the state $2^{-1}(b_a^{\dagger\,2} - b_b^{\dagger\,2})|0,0\rangle = 2^{-1/2}(|2,0\rangle - |0,2\rangle)$.

We note that Eq. (S11) represent a non local set of transformations. Indeed the beam splitter mixes the modes, so that a particle in mode a does not remain just in subspace a. Hence, in path notation the action of the beam splitter is non local.

Starting from the Fock state written in particle notation, the action of the beam splitter on the modes (a, b) is:

$$|a\rangle_l \rightarrow \frac{1}{\sqrt{2}}(|a\rangle_l + |b\rangle_l), \quad |b\rangle_l \rightarrow \frac{1}{\sqrt{2}}(|a\rangle_l - |b\rangle_l), \tag{S12}$$

with $l = 1, 2$. The transformation described by (S12) is local in the representation: particle l which identify the Hilbert space of the qudit remains the same before and after the transformation. The beam splitter, a linear optical element, cannot make

particles interact but merely mixes the modes. Transformation (S12) applied to a Fock state input (S10) generates an output state $2^{-1/2}(|a\rangle_1|a\rangle_2 - |b\rangle_1|b\rangle_2)$, which is the equivalent in particle notation of $2^{-1/2}(|2,0\rangle - |0,2\rangle)$.

Tritter is local in particle and non local in path. Here we extend the results for a multimode beam splitter, in particular we focus on the three-mode equivalent of the beam splitter, the tritter. The three-particle Fock state can be written in particle notation by considering all the permutations of three particles in three modes:

$$|1,1,1\rangle \to \frac{1}{\sqrt{6}}(|a\rangle_1|b\rangle_2|c\rangle_3 + |a\rangle_1|c\rangle_2|b\rangle_3 + |b\rangle_1|a\rangle_2|c\rangle_3 + |b\rangle_1|c\rangle_2|a\rangle_3 + |c\rangle_1|a\rangle_2|b\rangle_3 + |c\rangle_1|b\rangle_2|a\rangle_3) = |\{a,b,c\}\rangle_{123} \quad \text{(S13)}$$

This state is clearly separable in path notation and non-factorizable in particle notation and thus (particle) entangled. Now we prove that the unitary operation performed by the tritter is local in particle notation and non-local in path notation. The tritter matrix $\mathcal{U}^{(3)}$ has diagonal elements $(\mathcal{U}^{(3)})_{i,i} = 3^{-1/2}$ and off-diagonal elements $(\mathcal{U}^{(3)})_{i,j} = 3^{-1/2}e^{\imath 2\pi/3}$ with $i \neq j$. It operates on the creation operator so that $b_k^\dagger = \sum_i (\mathcal{U}^{(3)})_{i,k} a_k^\dagger$ holds. Applying the operation over the three-particle Fock state leads to:

$$|1,1,1\rangle \to \left(-\frac{1}{6\sqrt{3}} + \frac{i}{6}\right)(|3,0,0\rangle + |0,3,0\rangle + |0,0,3\rangle) + \left(\frac{1}{2\sqrt{3}} + \frac{i}{2}\right)|1,1,1\rangle. \quad \text{(S14)}$$

Analogously to the case of the beam splitter, the transformation over the creation operators is non-local in the path notation: indeed a particle in the Hilbert space defined by mode a is transformed in a linear superposition of particles in all the other modes. This property can be generalized to the case of an N-mode multiport interferometer.

We can define the tritter transformation in particle notation by considering that it mixes the modes:

$$\begin{aligned} |a\rangle_l &\to \tfrac{1}{\sqrt{3}}(|a\rangle_l + e^{2\imath\pi/3}|b\rangle_l + e^{2\imath\pi/3}|c\rangle_l), \\ |b\rangle_l &\to \tfrac{1}{\sqrt{3}}(e^{2\imath\pi/3}|a\rangle_l + |b\rangle_l + e^{2\imath\pi/3}|c\rangle_l), \\ |c\rangle_l &\to \tfrac{1}{\sqrt{3}}(e^{2\imath\pi/3}|a\rangle_l + e^{2\imath\pi/3}|b\rangle_l + |c\rangle_l), \end{aligned} \quad \text{(S15)}$$

with $l = 1,2,3$. We note again that these relations are local in this representation. If we apply the trasformation to the state (S13) we obtain:

$$\left(-\frac{1}{6\sqrt{3}} + \frac{i}{6}\right)(|a\rangle_1|a\rangle_2|a\rangle_3 + |b\rangle_1|b\rangle_2|b\rangle_3 + |c\rangle_1|c\rangle_2|c\rangle_3) + \left(\frac{1}{2\sqrt{3}} + \frac{i}{2}\right)|\{a,b,c\}\rangle_{123} \quad \text{(S16)}$$

which is completely equivalent to the result obtained with the path notation. Note that the parenthesis here represent all the possible permutation of the three particles. We again want to stress that this behavior is completely general: a multimode beam splitter is a linear optical elements which mixes the modes but does not make particle interact. As a consequence it can always be defined as a local operation over the particles, and a non local operation over the modes.

Phase shift transformation. A phase shift ϕ on mode k is represented by a transformation on the creation operators

$$b_j^\dagger = (1 + \delta_{j,k}(e^{-\imath\phi} - 1))a_j^\dagger, \quad \text{(S17)}$$

and in terms of particle notation:

$$|j\rangle_l \to (1 + \delta_{j,k}(e^{-\imath\phi} - 1))|j\rangle_l. \quad \text{(S18)}$$

The transformation in the two notations is equivalent and represent a local operation over a single mode.

Conclusion. The description of the overall interferometer is directly obtained from the previous considerations. Indeed, since the composition of local operations is still local, the action of the multimode MZI described in the main text is local in the particles, and thus cannot generate particle entanglement in the state. The results shown in the main text which describe a quantum advantage in the simultaneous estimation of multiple parameters is directly connected to the particle quantum correlations present in the input state. The calculations were performed using the path notation, in which creation operators and interferometric transformation are naturally defined. The quantum advantage is given by the particle entanglement in the input source. Indeed, in particle notation the interferometer is made by a series of local operations as considered by the theoretical analysis.

SUPPLEMENTARY NOTE 5: THE ESTIMATOR FOR THE ADAPTIVE PROTOCOL

The chosen estimator is essentially a Maximum likelihood estimator, with likelihood function $\mathcal{L}(\boldsymbol{\phi})$:

$$\mathcal{L}(\boldsymbol{\phi}) = \log\left[p^{(\alpha)}(\boldsymbol{\phi})\prod_{k=1}^{k_{\max}} p(k|\boldsymbol{\phi})^{n_k}\right]. \quad \text{(S19)}$$

Here, $p^{(\alpha)}(\boldsymbol{\phi})$ represents the knowledge on the parameters, $p(k|\boldsymbol{\phi})$ is the conditional probability of outcome k, and n_k is the number of occurrence of outcome k. At each step, the distribution $p^{(\alpha)}(\boldsymbol{\phi})$ is a Gaussian distribution:

$$p^{(\alpha)}(\boldsymbol{\phi}) = \prod_{i=1}^{2} \frac{1}{\sqrt{2\pi\sigma_i^{(\alpha)\,2}}} e^{-\frac{(\phi_1-\phi_i^{(\alpha)})^2}{(2\sigma_i^{(\alpha)\,2})}} , \tag{S20}$$

being $\phi_i^{(\alpha)}$ and $\sigma_i^{(\alpha)}$ the estimated value and the error on the parameter i obtained at step $\alpha - 1$ respectively.

SUPPLEMENTARY NOTE 6: ANALYTICAL EXPRESSION FOR THE FISHER INFORMATION MATRIX FOR THE 3-MODE MACH-ZEHNDER INTERFEROMETER

The expressions for the Fisher information matrix (FIM) with a $|1,1,1\rangle$ input state evolving in the 3-mode interferometer and measured with a photon-counting apparatus can be obtained from the definition of Eq. (3) in the main text. The probabilities $p(x|\boldsymbol{\lambda})$ of the general definition in this case are the probabilities $p_{m,n,q}(\boldsymbol{\phi})$ of observing an output state with respectively (m,n,q) photons (with $m+n+q=3$) on the three output modes after the overall interferometer transformation $\hat{U} = \hat{U}^{(3)}\hat{U}(\boldsymbol{\phi})\hat{U}^{(3)}$. The output probabilities can be derived as $p_{m,n,q}(\boldsymbol{\phi}) = |\langle m,n,q|\hat{U}|1,1,1\rangle|^2$. Finally, the FIM is obtained by applying Eq. (3).

We obtain in the 3-mode, two-phase scenario the following results for the elements of the 2×2 FIM matrix:

$$F_{11} = \frac{4}{9}\left[6\frac{N_A(\phi_1,\phi_2,-\frac{\pi}{3})}{D_A(\phi_1,\phi_2)} + 6\frac{N_A(\phi_1,\phi_2,\frac{\pi}{3})}{D_A(\phi_2,\phi_1)} + 4\frac{N_B^2(\phi_1,\phi_2)}{D_B(\phi_1,\phi_2)} + \frac{N_C^2(\phi_1,\phi_2)}{D_C(\phi_1,\phi_2)}\right]; \tag{S21}$$

$$F_{12} = F_{21} = \frac{4}{9}\left[6\frac{N_D(\phi_1,\phi_2)}{D_A(\phi_1,\phi_2)} + 6\frac{N_D(\phi_2,\phi_1)}{D_A(\phi_2,\phi_1)} + 4\frac{N_B(\phi_2,\phi_1)N_B(\phi_1,\phi_2)}{D_B(\phi_1,\phi_2)} + \frac{N_C(\phi_2,\phi_1)N_C(\phi_1,\phi_2)}{D_C(\phi_1,\phi_2)}\right]; \tag{S22}$$

$$F_{22} = \frac{4}{9}\left[6\frac{N_A(\phi_2,\phi_1,\frac{\pi}{3})}{D_A(\phi_1,\phi_2)} + 6\frac{N_A(\phi_2,\phi_1,-\frac{\pi}{3})}{D_A(\phi_2,\phi_1)} + 4\frac{N_B^2(\phi_2,\phi_1)}{D_B(\phi_1,\phi_2)} + \frac{N_C^2(\phi_2,\phi_1)}{D_C(\phi_1,\phi_2)}\right]; \tag{S23}$$

where we defined the following functions:

$$N_A(x_1,x_2,\alpha) = 2\sin^2\left(3x_1 - \frac{3}{2}x_2\right)\left[\cos\left(3x_2+\alpha\right)-1\right]; \tag{S24}$$

$$N_B(x_1,x_2) = 2\left[\sin\left(2x_1 - x_2 - \frac{\pi}{3}\right) - \sin\left(3x_1\right) - \sin\left(3x_1-3x_2\right)\right] + \sin\left(x_1 - 2x_2 + \frac{\pi}{3}\right) + \sin\left(x_1 + x_2 + \frac{\pi}{3}\right); \tag{S25}$$

$$N_C(x_1,x_2) = \sin\left(3x_1\right) + \sin\left(3x_1-3x_2\right) + 2\sin\left(2x_1 - x_2 - \frac{\pi}{3}\right) + \sin\left(x_1 - 2x_2 + \frac{\pi}{3}\right) + \sin\left(x_1 + x_2 + \frac{\pi}{3}\right); \tag{S26}$$

$$N_D(x_1,x_2) = 4\cos\left(\frac{3}{2}x_1 - \frac{\pi}{3}\right)\cos\left(\frac{3}{2}x_2 + \frac{\pi}{3}\right)\sin\left(3x_1 - \frac{3}{2}x_2\right)\sin\left(\frac{3}{2}x_1 - 3x_2\right); \tag{S27}$$

$$D_A(x_1,x_2) = 2\cos\left(3x_1 + \frac{\pi}{3}\right) + 2\cos\left(3x_2 - \frac{\pi}{3}\right) + 2\cos\left(3x_1 - 3x_2 - \frac{\pi}{3}\right) - 3; \tag{S28}$$

$$\begin{aligned} D_B(x_1,x_2) = 21 + 12&\left[\cos\left(x_1 - 2x_2 - \frac{2\pi}{3}\right) - \cos\left(2x_1 - x_2 - \frac{\pi}{3}\right) + \cos\left(x_1 + x_2 - \frac{2\pi}{3}\right)\right] + \\ &+ 8\left[\cos\left(3x_1\right) + \cos\left(3x_2\right) + \cos\left(3x_1 - 3x_2\right)\right]; \end{aligned} \tag{S29}$$

$$\begin{aligned} D_C(x_1,x_2) = 3&\left[2 + \cos\left(2x_1 - x_2 - \frac{\pi}{3}\right) + \cos\left(x_1 - 2x_2 + \frac{\pi}{3}\right) + \cos\left(x_1 + x_2 + \frac{\pi}{3}\right)\right] + \\ &+ \cos\left(3x_1\right) + \cos\left(3x_2\right) + \cos\left(3x_1 - 3x_2\right). \end{aligned} \tag{S30}$$

The results for a 4-mode interferometer with two phases can be obtained by adopting the same procedure described above.

[S1] S.M. Kay, Fundamentals of Statistical Signal Processing:Estimation Theory (Prentice Hall, New Jersey, 1993),Chap. 3.
[S2] S.L. Braunstein and C.M. Caves, *Phys. Rev. Lett.* **72**, 3439 (1994).
[S3] L. Pezzè and A. Smerzi, in *Atom Interferometry*, Proceedings of the International School of Physics Enrico Fermi, Course 188, edited by G. M. Tino and M. A. Kasevich (Società Italiana di Fisica, Bologna and IOS Press, Amsterdam), (to be published).
[S4] L. Pezzè and A. Smerzi, *Phys. Rev. Lett.* **102**, 100401 (2009).
[S5] V. Giovannetti, S. Lloyd and L. Maccone, *Phys. Rev. Lett.* **96**, 010401 (2006).
[S6] F. Benatti, R. Floreanini, U. Marzolino *Annals of Physics* **325**, 4, 924-935 (2010).
[S7] R. Demkowicz-Dobrzański, M. Jarzyna, J. Kolodński *Progress in Optics* **60**, 345-435 (2015).
